# Effects of Selenium-Enriched Yeast on Performance, Egg Quality, Antioxidant Balance, and Egg Selenium Content in Laying Ducks

**DOI:** 10.3389/fvets.2020.00591

**Published:** 2020-09-04

**Authors:** Xiufen Zhang, Lu Tian, Shuangshuang Zhai, Zhenping Lin, Huiyong Yang, Junpeng Chen, Hui Ye, Wence Wang, Lin Yang, Yongwen Zhu

**Affiliations:** ^1^Guangdong Provincial Key Laboratory of Animal Nutrition and Regulation, College of Animal Science, South China Agricultural University, Guangzhou, China; ^2^College of Animal Science, Yangtze University, Jingzhou, China; ^3^Institute of Baisha Livestock and Poultry Protospecies Research, Shantou, China; ^4^Key Laboratory of Feed Biotechnology, Ministry of Agriculture, Beijing, China

**Keywords:** antioxidant balance, egg selenium content, laying ducks, selenium-enriched yeast, selenium supplementation

## Abstract

This study investigated the effects of dietary selenium-enriched yeast (Se yeast) supplementation on the laying performance, egg quality, plasma antioxidant balance, and egg selenium (Se) content in laying Longyan ducks. A total of 480 32-week-old ducks were randomly divided into four dietary treatments, each consisting of six replicates of 20 ducks. The dietary treatments were a control basal diet and basal diets with supplementation of 0.05, 0.15, and 0.25 mg Se/kg via Se yeast. The analyzed Se contents of the four diets were 0.15, 0.21, 0.36, and 0.43 mg Se/kg, respectively. Dietary Se yeast supplementation had no apparent effects on laying performance and egg quality (*p* > 0.05), but it improved the antioxidant balance of ducks, as inferred by greater glutathione peroxidase and catalase activities, and decreased the malondialdehyde content in plasma of ducks (*p* < 0.05). It was suggested that the Se content in the basal diet containing 0.15 mg/kg of Se requirement is adequate for productive performance, but not for the antioxidant balance of laying ducks. Besides that, the Se contents in the yolk, albumen, and whole egg increased linearly as the Se supplementation levels increased. With more feeding days, the Se contents in the yolk and whole egg from non-Se-yeast-supplemented ducks increased linearly (*p* < 0.05), while those from Se-yeast-supplemented ducks showed a quadratic relationship (*p* < 0.05). In conclusion, the Se content of the basal diet at 0.15 mg/kg was adequate for laying performance and egg quality traits in laying ducks. Dietary Se yeast supplementation is beneficial to improve the antioxidant balance of laying ducks and increase the Se deposition in eggs for producing Se-enriched eggs. Based on the quadratic model or the quadratic broken-line model analyses, supplemental 0.19 mg Se/kg via Se yeast, with a total equivalent of 0.34 mg Se/kg in the diet, could provide the optimum antioxidant balance in laying ducks. Dietary supplementation of 0.25 mg Se/kg via Se yeast, with a total equivalent of 0.40 mg Se/kg in the diet, could lead to achieving the desired Se content in the whole egg.

## Introduction

As a key component of glutathione peroxidase and selenoproteins, selenium (Se) is essential for the execution of oxidoreductase functions and plays a crucial role in various biological processes of animals, such as the fertilization capacity of spermatozoa, embryonic and post-natal development, and immune responses (1–5). Therefore, Se-enriched animal products are gaining popularity for improving the Se health status of their human consumers. A fair number of studies tried to produce Se-enriched animal products via feed-based nutritional interventions to increase Se deposition in meat, egg, and milk (6–10). For example, Se deposition in egg yolk and albumen rose as dietary Se supplement levels increased, regardless of the Se source used (3, 11, 12). Other reports, however, did not find any elevated Se deposition in eggs from hens that were fed an insufficient Se-supplemented diet below the requirement of laying hens (13–15). Previous studies on laying hens and quails showed that those given organic Se sources, because of its higher bioavailability, received greater egg Se deposition compared with those given inorganic Se sources (16–18). Organic selenium-enriched yeast (Se yeast), containing 50–75% selenomethionine (SeMet), has been widely used in poultry diets (19–21). SeMet could be incorporated into proteins in place of Met and then enter into a long-term body pool, with a slower turnover rate than sodium selenite (SS), resulting in greater Se deposition in the eggs than that obtained by SS (22–24). For example, 34.26–37.12 μg Se per egg was provided by hens fed a Se-yeast-supplemented diet for 84 days, values 67.94–67.96% greater than those available from a SS-supplemented diet (25). Furthermore, it is speculated that egg deposition efficiency mainly depends on dietary Se level and Se sources, as well as the experimental feeding time. Additionally, there can be a wide variation in Se deposition efficiency in eggs among different domestic avian species. The Se contents of egg albumen and yolk in ducks were higher than those found in chicken, turkeys, and geese when these birds were offered the same feed type and schedule (26). Most studies of egg Se deposition have focused on laying hens that received diets with Se supplementation, leaving ducks rarely investigated in this respect (27–29). Accordingly, here we tested to investigate the effects of Se supplementation above its basal requirement, using Se yeast as an organic Se source, on laying performance, egg quality, egg Se deposition, and plasma antioxidant indices in ducks.

## Materials and Methods

The protocol was reviewed and approved by the Animal Care and Use Committee of South China Agricultural University.

Longyan duck (*Anas platyrhynchos*) is a local breed of ducks for the dual production of eggs and meat in South China. A total of 480 32-week-old Longyan duck layers, during the middle–late laying period, with a similar initial laying rate (56.28 ± 1.97 %), were randomly divided into four treatments, each consisting of six replicates of 20 ducks. Two duck layers were housed in a single wire cage (40 cm length × 40 cm width × 50 cm height) equipped with three ladders and allowed *ad libitum* access to tap water. The 80-g feed was provided twice daily at 08:00 and 16:00 h. The corn–soybean meal basal diet served as the control diet; it was formulated to satisfy the basic nutrient requirements for Longyan laying ducks, containing 0.15 mg/kg of Se (Table 1). The three dietary Se treatments were formulated by supplementing the basal diet with 0.05, 0.15, or 0.25 mg/kg of Se via Se yeast (2,000 mg Se/kg Se yeast, Boshan Biological Feed Co., Ltd., Guangzhou, China). The total analyzed Se contents of the four diets were thus 0.15, 0.21, 0.36, and 0.43 mg Se/kg, respectively. The experimental time lasted for 28 days. During this period, eggs were collected from egg trays and the total egg weight of each replicate was recorded daily. The feed-to-egg ratio was calculated as the egg mass divided by feed intake. Two eggs per replicate were randomly selected, and the albumen and yolk were separated for Se assay. Another two eggs were broken and their egg albumen and yolk were mixed to prepare the whole egg samples. The Se contents in egg albumen, egg yolk, and the whole egg samples were measured at days 1, 7, 14, 21, and 28 of the experimental period. The experiment was conducted in a tropical area (Shantou City, China; 116° E longitude, 231° N latitude). The birds were kept in a semi-open laying house at 30 ± 2°C with 60–65% relative humidity and maintained on a 16-h light and 8-h dark photoperiod cycle.

**Table 1:** Ingredients and nutrient levels of the basal diet (as-fed basis).

**Ingredients**	**Composition, %**	**Nutrient**	**Nutrient levels**
Corn	52.8	ME, MJ/kg	10.5
Soybean meal	23.0	Crude protein, %	17.0
Wheat bran	11.8	Calcium, %	3.50
Fish meal	2.00	Available phosphorus, %	0.350
Limestone	8.30	Methionine, %	0.402
Calcium hydrophosphate	0.70	Methionine + cystine, %	0.673
Sodium chloride	0.20	Lysine, %	0.956
DL-Methionine	0.14	Selenium^a^, mg/kg	0.15
L-Lysine hydrochloride	0.060		
Premix^b^	1.00		
Total	100		

a *Measured value of Se level; levels of other nutrients were calculated values. The total analyzed Se contents of the four treatment diets were 0.15, 0.21, 0.36, and 0.43 mg Se/kg, respectively*.

b *Supplied per kilogram of diet: 16,000 IU of vitamin A, 4,000 IU of vitamin D3, 40 mg of vitamin E, 8 mg of vitamin K3, 4 mg of vitamin B1, 12 mg of vitamin B2, 8 mg of vitamin B6, 0.04 mg of vitamin B12, 0.2 mg of D-biotin, 24 mg of D-pantothenic acid, 2 mg of folic acid, 80 mg of nicotinamide, 50 mg of choline chloride, 6.4 mg of Cu, 70 mg of Fe, 80 mg of Zn, 96 mg of Mn, 0.56 mg of I, 0.32 mg of Co, 0.15 mg of Se*.

### Egg Quality

At the end of the experiment, 24 eggs per treatment (four eggs per replicate) were randomly selected for egg quality measurement. The eggs were weighed individually, and then their respective length and width were recorded with an egg form coefficient measuring instrument (FHK Fujihira Industry Co., Ltd., Tokyo, Japan) before breaking. Eggshell strength was measured with an egg force reader (Tenovo International Co., Ltd., Beijing, China), and eggshell thickness (average of three locations around the eggshell) was determined with an eggshell thickness gauge (FHK Fujihira Industry Co., Ltd., Tokyo, Japan). Yolk color, albumen height, and Haugh unit were all assayed by the egg analyzer (ORKA Food Technology Co. Ltd., Ramat HaSharon, Israel). The yolks were detached with an egg separator, weighed, and expressed as a percentage of egg weight. The weight of dried eggshells with their shell membranes was also weighed and likewise expressed as a percentage of egg weight. The albumen weight was calculated by subtracting the yolk and shell weight from the total egg weight, expressed also as a percentage of egg weight.

### Selenium Assay

The Se content assay was performed as described by Meng et al. (28). In brief, a given homogenized egg sample or feed sample was mixed with HNO_3_-HClO_4_ (4:1) and heated until white fumes appeared; at this point, hydrochloric acid solution was added to digest it. Next, the digested sample was cooled down and diluted with ultrapure water for its measurement with an atomic fluorescence spectrophotometer (SK-2003A, Beijing Jinsuokun Technology Development Co., Ltd., Beijing, China).

### Plasma Biochemical Assay

On day 28, one duck per replicate was randomly selected for drawing of blood sample from its wing vein. These blood samples were centrifuged at 3,000 rpm for 10 min, and the ensuing plasma samples were kept in Eppendorf tubes at −20°C until their analysis. Total protein (TP), albumin (ALB), alanine transaminase (ALT) enzyme, and aspartate transaminase (AST) enzyme levels were determined by an automatic biochemical analyzer (BS-5800M, Mindray Bio-Medical Electronics Co. Ltd., Shenzhen, China). Globulin (GLB) was calculated by subtracting ALB from TP from which the ALB/GLB ratio was then obtained. Total antioxidant capability (TAC), superoxide dismutase (SOD), glutathione peroxidase (GSH-Px), catalase (CAT), and malondialdehyde (MDA) in plasma were determined by following the manufacturer's instructions of the respective assay kits (Nanjing Jiancheng Bioengineering Institute, Nanjing, China), and then the MDA/TAC ratio was calculated as described by Attia et al. (30).

### Statistical Analysis

Data were analyzed by one-way ANOVA using the PROC GLM procedure of the Statistical Analysis system v9.2 (SAS Inst. Inc., Cary, NC, USA). Orthogonal polynomial contrasts were used to determine the form of the effect (linear, quadratic, or cubic) as a function of higher Se levels in the diet or more experimental days. Data on laying rate, yolk proportion, albumen proportion, and eggshell proportion were transformed into arc sine values before statistical analysis. Each replicate served as the experimental unit for all statistical analyses. Data were deemed significant at *p* < 0.05. The regression of antioxidant indices was estimated by quadratic model or quadratic broken-line model analyses to discern the optimum antioxidant balance. The quadratic regression model is *Y* = *A* + *BX* + *CX*^2^, where *Y* means the antioxidant indices in plasma, and Se supplementation from Se yeast was estimated when the concentration of antioxidant indices reached the maximum response. The quadratic broken-line model for regression is *Y* = *L* + *U* × (*R* – *X*)^2^, where *L* is the plateau at values of *x* > *R, R* is the abscissa of the breakpoint, and the value of (*R* – *X*) is zero at values of *X* > *R* (31). *Y* means the antioxidant indices in plasma, *L* means the concentration of antioxidant indices at the plateau, and *R* means the Se supplementation from Se yeast. The plateau breakpoint for each Se response curve was determined by straight or quadratic broken-line model analyses to discern the optimum Se content in the whole egg (31). The straight broken-line model is *Y* = *L* + *U* × (*R* – *X*), and the quadratic broken-line model is *Y* = *L* + *U* × (*R* – *X*)^2^, where *Y* means the Se content in eggs, *L* means the Se content at the plateau, and *R* means the experimental day.

## Results

### Laying Performance

Dietary Se yeast supplementation had negligible effects (*p* > 0.05) on the laying rate, egg weight, egg mass, and feed/egg ratio in laying ducks (Table 2).

**Table 2:** Effect of dietary Se yeast supplementation on the laying performance of Longyan laying ducks (*n* = 6)^a^.

**Items**	**Supplemented Se level from Se yeast, mg/kg**				**SEM**	***P*****-values**			
	**0**	**0.05**	**0.15**	**0.25**		**Treatment**	**Linear**	**Quadratic**	**Cubic**
Laying rate, %	59.2	55.8	54.6	58.8	2.40	0.466	0.973	0.120	0.893
Egg weight, g	56.6	56.7	57.1	57.1	0.49	0.841	0.409	0.787	0.832
Egg mass, g/day/bird	33.5	31.6	31.2	33.6	1.40	0.501	0.877	0.138	0.872
Feed: egg ratio	5.02	5.29	5.36	4.97	0.222	0.528	0.794	0.153	0.909

*Se yeast, selenium-enriched yeast*.

*SEM, standard error of the mean*.

a *Means were calculated using six replicates per treatment (20 ducks per replicate)*.

### Egg Quality

Dietary Se yeast supplementation had no apparent effect (*p* > 0.05) on the egg quality of laying ducks, in terms of the yolk proportion, albumen proportion, eggshell proportion, egg shape index, eggshell strength, eggshell thickness, albumen height, Haugh unit, and egg yolk color (Table 3).

**Table 3:** Effect of dietary Se yeast supplementation on the egg quality of Longyan laying ducks (*n* = 6)^a^.

**Items**	**Supplemented Se level from Se yeast, mg/kg**				**SEM**	***P*****-values**			
	**0**	**0.05**	**0.15**	**0.25**		**Treatment**	**Linear**	**Quadratic**	**Cubic**
Yolk proportion, %	30.1	30.9	31.1	30.7	0.89	0.856	0.677	0.470	0.882
Albumen proportion, %	54.5	50.2	54.2	55.3	1.21	0.578	0.378	0.406	0.440
Eggshell proportion, %	15.1	16.0	14.7	14.1	0.70	0.260	0.103	0.512	0.287
Egg shape index	73.9	74.1	72.4	72.1	1.09	0.454	0.128	0.923	0.575
Eggshell strength, kg/cm^2^	4.15	4.32	3.94	4.01	0.178	0.407	0.257	0.859	0.204
Eggshell thickness, mm	0.341	0.342	0.342	0.343	0.006	0.538	0.362	0.414	0.380
Albumen height, mm	6.02	6.26	6.38	5.85	0.285	0.550	0.640	0.163	0.936
Haugh unit	77.9	78.9	80.5	76.2	2.04	0.510	0.608	0.157	0.702
Egg yolk color	4.46	4.65	4.20	4.55	0.204	0.448	0.812	0.414	0.157

*Se yeast, selenium-enriched yeast; SEM, standard error of the mean*.

a *Means were calculated using six replicates per treatment (four eggs per replicate)*.

### Plasma Biochemical and Antioxidant Indices

Dietary Se yeast supplementation had no discernable impact (*p* > 0.05) on the TP, ALB, and GLB contents and ALB/GLB ratio, as well as the activities of ALT, AST, and SOD in the duck's plasma (Table 4). However, in response to increasing Se supplementation from Se yeast, the CAT activity increased in a linear way (*r*^2^ = 0.472, *p* < 0.001), while GSH-Px activity was elevated in both linear (*r*^2^ = 0.296, *p* < 0.001) and quadratic manner (*r*^2^ = 0.955, *p* < 0.001). For TAC, it was enhanced in a linear (*r*^2^ = 0.168, *p* < 0.001), quadratic (*r*^2^ = 0.861, *p* < 0.001), and cubic manner (*r*^2^ = 0.955, *p* < 0.001) with increasing Se supplementation from Se yeast. By contrast, negative linear (*r*^2^ = 0.453, *p* < 0.001), quadratic (*r*^2^ = 0.950, *p* < 0.001), and cubic relationship (*r*^2^ = 0.974, *p* < 0.001) was detected between the MDA content in plasma and dietary-supplemented Se level from Se yeast. The MDA/TAC ratio was decreased in a linear (*r*^2^ = 0.390, *p* < 0.001), quadratic (*r*^2^ = 0.894, *p* < 0.001), and cubic manner (*r*^2^ = 0.922, *p* < 0.05) with increasing Se supplementation from Se yeast. The highest GSH-Px and TAC activities and the lowest MDA content in plasma characterized those ducks fed a diet with 0.15 mg/kg Se supplementation from Se yeast. According to the regression (Table 5), the Se supplementation from Se yeast ranged from 0.11 to 0.32 mg/kg, and the average value was 0.19 mg/kg when the concentration of antioxidant indices reached the maximum response.

**Table 4:** Effect of dietary Se yeast supplementation on the biochemical and the antioxidant indices in plasma of Longyan laying ducks (*n* = 6)^a^.

**Items**	**Supplemented Se level from Se yeast, mg/kg**				**SEM**	***P*****-values**			
	**0**	**0.05**	**0.15**	**0.25**		**Treatment**	**Linear**	**Quadratic**	**Cubic**
TP, mg/ml	5.54	5.59	5.20	5.22	0.176	0.424	0.177	0.755	0.458
ALB, mg/ml	1.54	1.50	1.37	1.34	0.163	0.846	0.433	0.857	0.917
GLB, mg/ml	4.00	4.10	3.83	3.88	0.278	0.941	0.675	0.926	0.679
ALB/GLB ratio	0.407	0.378	0.427	0.350	0.074	0.991	0.845	0.855	0.964
ALT, U/L	3.07	3.13	3.02	3.17	0.303	0.993	0.911	0.863	0.819
AST, U/L	2.73	2.75	2.36	2.05	0.347	0.576	0.175	0.863	0.813
GSH-Px, mmol/ml	173^c^	274^b^	365^a^	272^b^	6.5	<0.001	<0.001	<0.001	0.393
SOD, U/ml	22.2	22.8	25.1	26.3	4.61	0.910	0.478	0.956	0.939
CAT, U/ml	1.71^c^	2.51^bc^	3.28^ab^	3.90^a^	0.376	0.003	<0.001	0.470	0.712
TAC, U/ml	3.53^c^	5.65^b^	10.6^a^	5.61^b^	0.251	<0.001	<0.001	<0.001	<0.001
MDA, nmol/ml	32.8^a^	23.4^b^	18.8^c^	22.5^b^	0.38	<0.001	<0.001	<0.001	<0.001
MDA/TAC ratio	9.55^a^	4.16^b^	1.78^b^	4.03^c^	0.373	<0.001	<0.001	<0.001	0.0152

*Se yeast, selenium-enriched yeast; SEM, standard error of the mean; TP, total protein; Alb, albumin; Glb, globulin; ALT, alanine transaminase enzyme; AST, aspartate transaminase enzyme; GSH-Px, glutathione peroxidase; SOD, superoxide dismutase; CAT, catalase; TAC, total antioxidant capability; MDA, malondialdehyde*.

a *Means were calculated using six replicates per treatment (one bird per replicate)*.

a, b, c *Mean values within a row with different letters were significantly different (p < 0.05)*.

**Table 5:** Estimated change of antioxidant indices in plasma with the Se supplementation from Se yeast (*n* = 6)^a^.

**Variation**	**Equation of regression**	**Estimated Se supplementation from Se yeast (mg/kg)**	**Estimated maximum response**	***P*-values**	***R*^2^**
Quadratic regression equation^b^					
GSH-Px, mmol/ml	*Y* = 170.6 + 2581.7*X* – 8693.9*X*^2^	0.15	362	<0.001	0.955
CAT, U/ml	*Y* = 1.77 + 13.78*X* – 21.32*X*^2^	0.32	3.99	<0.001	0.486
TAC, U/ml	*Y* = 2.88 + 96.95*X* – 340.19*X*^2^	0.14	9.79	<0.001	0.861
MDA/TAC ratio	*Y* = 9.16 – 101.36*X* + 325.78*X*^2^	0.16	1.27	<0.001	0.894
Quadratic broken-line regression equation^c^					
GSH-Px, mmol/ml	*Y* = 318.4 – 11565.1 × (0.11 – *X*)^2^	0.11	318	<0.001	0.732
CAT, U/ml	*Y* = 3.99 – 21.32 × (0.32 – *X*)^2^	0.32	3.99	<0.001	0.486
TAC, U/ml	*Y* = 8.03 – 195.3 × (0.15 – *X*)^2^	0.15	8.03	<0.001	0.512

*Se yeast, selenium-enriched yeast; GSH-Px, glutathione peroxidase; CAT, catalase; TAC, total antioxidant capability; MDA, malondialdehyde*.

a *Means were calculated using six replicates per treatment (one bird per replicate)*.

b *The quadratic model for regression is Y = A + BX + CX^*2*^. Y means the antioxidant indices in plasma, and the Se supplementation from Se yeast was estimated when the concentration of the antioxidant indices reached the maximum response*.

c *The quadratic broken-line model for regression is Y = L + U × (R – X)^*2*^, where L is the plateau at values of X > R, R is the abscissa of the breakpoint, and the value of (R – X) is zero at values of X > R. Y means the antioxidant indices in plasma, L means the concentration of antioxidant indices at the plateau, and R means the Se supplementation from Se yeast*.

### Se Contents

**Figure 1:**
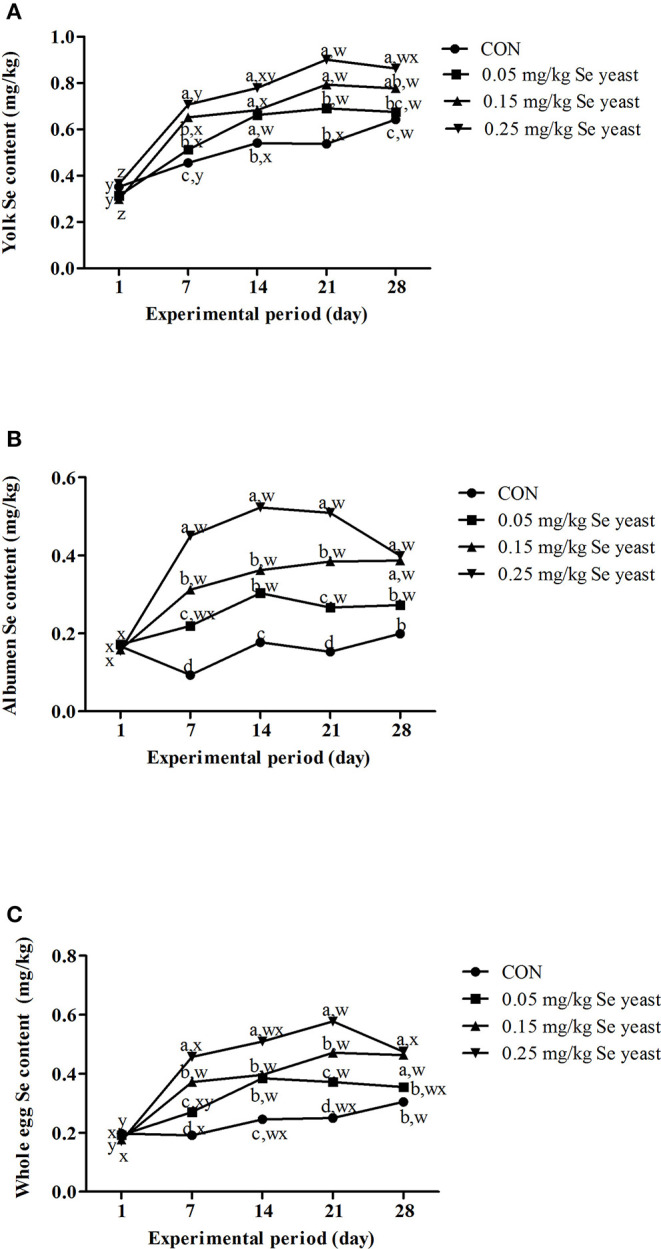
**(A)** Effect of dietary Se yeast supplementation on the dynamic change of yolk Se contents (mg/kg, fresh weight basis) during the 28-d feeding period. **(B)** Effect of dietary Se yeast supplementation on the dynamic change of albumen Se contents (mg/kg, fresh weight basis) during the 28-d feeding period. **(C)** Effect of dietary Se yeast supplementation on the dynamic change of the whole egg Se contents (mg/kg, fresh weight basis) during the 28-d feeding period. CON = the basal diet with 0.15 mg Se/kg from premix, 0.05 mg/kg Se yeast = the basal diet plus 0.05 mg/kg of Se from Se yeast, 0.15 mg/kg Se yeast = the basal diet plus 0.15 mg/kg of Se from Se yeast, 0.25 mg/kg Se yeast = the basal diet plus 0.25 mg/kg of Se from Se yeast. Values on the same day with different superscripts (a, b, c, d) are significantly different at *p* < 0.05. Values on the same line with different superscripts (w, x, y, z) are significantly different at *p* < 0.05. Egg Se contents for each treatment data on the same experimental day are means of 6 replicates (2 eggs per replicate).

With a greater Se supplementation from Se yeast, the Se contents of the yolk, albumen, and whole egg all increased linearly (*p* < 0.05) on days 7, 14, 21, and 28 (Figures 1A–C). With more time (*i*. *e*., days of the experiment), the Se contents of the yolk and the whole egg increased linearly (*p* < 0.05) in the control group (Figures 1A,C), whereas those of the yolk, albumen, and whole egg all increased quadratically (*p* < 0.05) for the Se-yeast-supplementation treatments (*i*. *e*., the basal diet plus 0.05, 0.15, and 0.25 mg Se/kg from Se yeast, Figures 1A–C). Based on the broken-line analysis, the estimated experimental days for reaching the plateaued response of Se content in the whole egg declined as the supplemented Se level from Se yeast increased (Table 6). The maximum Se contents of whole eggs from ducks receiving diets with 0.05, 0.15, and 0.25 mg/kg Se supplementation via Se yeast were estimated to be 0.37, 0.44, and 0.53 mg/kg on days 18, 13, and 12, respectively (Table 6).

**Table 6:** Estimated change of Se content in eggs with the increase in experimental days.

**Variation**	**Supplemented Se level from Se yeast, mg/kg**	**Regression equations^a^**	**Estimated days**	**Estimated Se content in eggs, mg/kg**	***P*-values**	***R*^**2**^**
Yolk Se content (mg/kg)	0	*Y* = 0.71 – 0.0095 × (36.0 – *X*)	36	0.71	<0.001	0.510
	0.05	*Y* = 0.68 – 0.0012 × (18.9 – *X*)^2^	19	0.68	<0.001	0.622
	0.15	*Y* = 0.75 – 0.0036 × (12.3 – *X*)^2^	13	0.75	<0.001	0.650
	0.25	*Y* = 0.87 – 0.0013 × (20.0 – *X*)^2^	20	0.87	<0.001	0.647
Albumen Se content (mg/kg)	0.05	*Y* = 0.28 – 0.00050 × (16.3 – *X*)^2^	17	0.28	0.018	0.492
	0.15	*Y* = 0.38 – 0.0012 × (14.4 – *X*)^2^	15	0.38	<0.001	0.805
	0.25	*Y* = 0.48 – 0.0043 × (9.7 – *X*)^2^	10	0.48	<0.001	0.704
Whole-egg Se contents (mg/kg)	0	*Y* = 0.33 – 0.0042 × (36.7 – *X*)	37	0.33	0.001	0.567
	0.05	*Y* = 0.37 – 0.00068 × (17.7 – *X*)^2^	18	0.37	0.001	0.650
	0.15	*Y* = 0.44 – 0.0018 × (12.9 – *X*)^2^	13	0.44	<0.001	0.724
	0.25	*Y* = 0.53 – 0.0029 × (11.8 – *X*)^2^	12	0.53	<0.001	0.877

a *The regression equation for the non-Se-yeast-supplementation group was the linear broken-line model Y = L + U × (R – X), while for the Se-yeast-supplementation treatment groups, it was the quadratic broken-line model Y = L + U × (R – X)^2^, where L is the plateau at values of X > R, R is the abscissa of the breakpoint, and the value of (R – X) is zero at values of X > R. Y means the Se content in eggs, L means the Se content at the plateau, and R means the experimental day*.

## Discussion

It is generally recognized that Se is an essential element underpinning reproductive performance in poultry. Dietary Se supplementation with different levels (0.10–1.00 mg/kg) and sources (SS, Se yeast, and nano-Se or Se-enriched probiotics) improves both the laying performance and the egg quality of hens and quails (8, 11, 12, 14, 28, 32). In our study, supplementation with 0.05–0.25 mg Se/kg in the form of Se yeast included in the basal diet of Longyan laying ducks had no significant effect on their laying performance. These results agree with studies of Shanma laying ducks and Hy-Line, Leghorn, and Brown Warren laying hens that were fed diets supplemented with Se sources varying from 0.08 to 0.50 mg Se/kg (25, 33–35). Besides that, it was reported that egg production was not significantly affected by the level (0.10–0.40 mg/kg) and the source of Se, and no significant effect on the interaction between these variables was observed either (36). The divergent results for the laying performance may be due to the differences in Se sources and levels, avian species, age, and rearing environmental conditions. For example, Longyan laying ducks, a local breed of ducks for the dual production of eggs and meat in South China, which were used in our experiment, had a relatively low laying performance compared to the commercial-strain hens during the mid–late laying period. Besides that, this experiment was conducted in a tropical area with high temperature and relative humidity, which resulted in a slight decrease in the overall egg production performance of birds during the experimental period. Concerning egg quality, Baylan et al. (8) showed that, for quail eggs, their eggshell weight and eggshell thickness increased with inorganic or organic Se supplementation (0.10–0.30 mg Se/kg from SS or Se yeast), but excessive dietary Se can impair eggshell quality, as revealed by the reduced eggshell thickness in ducks and the lower eggshell proportion and strength in hens (29, 35). Those significant differences on laying performance and egg quality by dietary Se treatments were not observed in our study, which could be mainly explained by poultry breed, Se content of the basal diet, Se sources, and the duration of the experiment. We thus speculated that the Se content of the basal diet at 0.15 mg/kg was adequate for laying performance and egg quality traits in ducks. Moreover, the 28-day experimental period might be too short to induce a significant effect on the laying performance and egg quality of the duck breed that we studied.

As a component of the GSH-Px enzyme and selenoproteins, Se can improve the antioxidant capacity and reduce the lipid peroxides (37). The beneficial effect of enhanced antioxidant activity was observed in chicken broilers, laying hens, and pigeons when fed diets containing 0.10–0.90 mg Se/kg supplementation, regardless of the Se sources used (SS, Se yeast, nano-Se, or DL-SeMet) (7, 15, 38–40). Jing et al. (15) demonstrated that, compared to inorganic sources, organic Se sources fostered a better antioxidant balance by increasing the activities of GSH–Px and SOD and decreasing the MDA content in plasma. We found that plasma GSH-Px and CAT activities in laying ducks fed Se-yeast-supplemented diets rose remarkably relative to layers fed with the basal diet only. Furthermore, plasma TAC activity was increased when the laying ducks were given Se-yeast-supplemented diets, a result that is consistent with other findings reported for laying hens (28). Additionally, dietary Se yeast supplementation reduced the plasma MDA content, pointing to less lipid peroxidation damage, which could reflect the greater antioxidant capacity in these ducks. By comparison with the results of laying performance and egg quality, it was implied that the basal diet with 0.15 mg Se/kg was adequate for productive performance but not for antioxidant balance. In addition, it was acknowledged that Se is key to the maintenance of various physiological and biochemical functions within a small amount, while an excessive dose may lead to harmful effects (41). The Se supplementation at 0.15 and 0.25 mg/kg diet via Se yeast improves the antioxidant balance of the laying ducks in our study, and the average value of Se supplementation level from Se yeast was 0.19 mg/kg for optimum antioxidant balance. Heat stress affects the growth performance in broilers and the laying performance in laying hens, leading to a compromising efficiency in poultry production (42–44). So far, the potential enhancement of antioxidant ability from organic Se sources has been applied to alleviate the negative effect on poultry production when this is subjected to environmental stress challenge (45, 46). For example, many studies in broilers showed that the dosage of Se, added at a range of 0.3–1.0 mg/kg to the diet, improved the growth performance of chicken and quails when exposed to heat stress, contributing to the improvement of immune function and the increased activities of glutathione, GSH-Px, and SOD (47–53). It is also implied that a greater amount of Se is required for enhancing selenium-dependent antioxidant enzyme activities and gene expression relative to the glutathione system to alleviate oxidative damage induced by heat stress in poultry (54–59). According to the positive result of antioxidant ability, we will further investigate the Se requirement as well as the effect of Se yeast supplementation in diets in duck layers under heat-stressed condition in our next study.

Se deficiency could impair human health, given its association with a weaker immune system and a greater risk of susceptibility to various diseases (37, 60). The mineral contents of eggs also reflected the nutrition and the health status of laying hens (61). So far, one of the effective ways to improve Se intake is to consume some functional food, such as Se-enriched egg, meat, and milk products (10, 27, 62). However, the Se content in ordinary eggs collected from the retail market is under detectable levels (41, 63). Many studies have demonstrated that organic Se supplementation of the diets of laying hens translates into greater Se content and Se deposition efficiency in their eggs compared to using inorganic Se (16, 17, 64, 65). Since ducks may have a greater Se transfer rate among poultry, they could be an easier source to obtain Se-enriched eggs from than hens, turkeys, and geese (26). For this reason, how the supplemental Se level from the Se yeast source above the Se requirement affects egg Se deposition in laying ducks was investigated in the present study. Our results showed that egg Se deposition in ducks increased as dietary Se yeast supplementation increased, corroborating previous studies of laying hens that received diets with Se supplementation levels of 0.20 to 1.00 mg Se/kg (11, 12, 66, 67). Moreover, the Se content of whole eggs could be achieved at 0.53 mg/kg in laying ducks fed a diet containing 0.25 mg Se/kg from Se yeast for 13 days, which was less than the 0.50 mg/kg in laying hens fed Se-yeast-supplemented diets containing 0.30 to 0.50 mg Se/kg for 28 days, though the basal diets in the above-mentioned condition already contained 0.15 mg/kg Se (25, 28, 68). Therefore, we suggest that Se deposition efficiency might be greater for laying ducks than for laying hens. Interestingly, the Se contents of eggs from laying ducks fed the Se-yeast-supplemented diets were increased quadratically as the feeding days increased. The days required to attain the maximum Se deposition in whole eggs reduced as dietary Se yeast supplementation increased. From the broken-line analyses, the days were shorter for duck layers fed diets with 0.25 or 0.15 mg Se/kg supplementation via Se yeast (12 or 13 days) to peak the Se content in a whole egg than that fed diet with 0.05 mg Se/kg supplementation via Se yeast or the control basal diet (18 or 37 days). Therefore, to produce duck Se-enriched eggs, we recommend feeding ducks a diet (containing 0.15 mg Se/kg) supplemented with additional 0.15–0.25 mg Se/kg via Se yeast for no more than 12–13 days.

## Conclusions

Our results showed that dietary Se yeast supplementation had no apparent effects on laying performance and egg quality, but it improved the glutathione peroxidase and catalase activities and decreased the malondialdehyde content in the plasma of ducks. Also, the Se contents in the egg yolk, albumen, and whole egg increased linearly as dietary Se levels increase. From the findings, the Se content of the basal diet at 0.15 mg/kg was adequate for laying performance and egg quality traits. Dietary Se yeast supplementation is beneficial to improve the antioxidant balance of laying ducks and increase Se deposition in eggs to produce Se-enriched eggs. Based on the quadratic model or the quadratic broken-line model analyses, supplemental 0.19 mg Se/kg via Se yeast, with a total equivalent of 0.34 mg Se/kg in the diet, could provide the optimum antioxidant balance in laying ducks. Dietary supplementation of 0.25 mg Se/kg via Se yeast, with a total equivalent of 0.40 mg Se/kg in the diet, could lead to achieving the desired Se content in the whole egg.

## Data Availability Statement

The original contributions presented in the study are included in the article/supplementary material, further inquiries can be directed to the corresponding author/s.

## Ethics Statement

The animal study was reviewed and approved by Animal Care and Use Committee of South China Agricultural University.

## Author Contributions

XZ designed this study, carried out the experiments and measurements, and drafted the manuscript. LT and SZ helped with the analysis of the samples. ZL, HYa, and JC assisted with the duck trial. HYe and WW helped with the data analysis. LY and YZ participated in the study's design, coordination, and manuscript writing. All authors read and approved the final manuscript.

## Conflict of Interest

The authors declare that the research was conducted in the absence of any commercial or financial relationships that could be construed as a potential conflict of interest.

## Abbreviations

Se: selenium
Se yeast: selenium-enriched yeast
SeMet: selenomethionine
SS: sodium selenite
TP: total protein
ALB: albumin
GLB: globulin
ALT: alanine transaminase
AST: aspartate transaminase
TAC: total antioxidant capability
SOD: superoxide dismutase
GSH-Px: glutathione peroxidase
CAT: catalase
MDA: malondialdehyde.
